# A census of laparoscopic and robotic urological practice: a survey of minimally invasive surgery department of the Brazilian Society of Urology

**DOI:** 10.1590/S1677-5538.IBJU.2018.0724

**Published:** 2019-09-02

**Authors:** Marcos Flávio Holanda Rocha, Rafael Ferreira Coelho, Anibal Wood Branco, Pedro Henrique de Oliveira Filgueira, Rômolo Guida

**Affiliations:** 1 Hospital Monte Klinikum, Fortaleza, CE, Brasil;; 2 Hospital Albert Einstein, São Paulo, SP, Brasil;; 3 Hospital Marcelino Champagnat, Curitiba, PR, Brasil;; 4 Hospital Federal dos Servidores do Estado, Rio de Janeiro, RJ, Brasil

**Keywords:** Education, Robotic Surgical Procedures, Urology

## Abstract

Minimally invasive urologic surgery has been developing in Brazil and now is a routine part of care in many regions and patients with different conditions benefit from it. Training in laparoscopic and robotic surgery has evolved and concerns exist both over the quality of surgical training and the practical effect on results of the urological training. This is an unprecedented study which undertook a census to determinate the current state of laparoscopic and robotic urological practice and to know the mains barriers to adequate practice in Brazil. In august 2017, surveys, consisting of an anonymous questionnaire with 15 questions, were sent via internet to the mailing list of the Brazilian Society of Urology (SBU). With these data, activities related to laparoscopy and robotic surgery of our urologists and the mains difficulties and barriers to practice laparoscopy and robotic surgery were evaluated. In our survey, 413 questionnaires were completed. Majority of the responders were currently working in the southeast region of Brazil (52.1%) and 75.5% of the surgeons performed laparoscopic surgery while, only 12.8%, robotic surgery. The lack of experience on the technique and the lack of equipment were the mains barriers and difficulties for not executing laparoscopic and robotic surgeries, respectively. Proper longitudinal training and access to good equipment in minimally invasive surgery are still barriers for urologists in our country.

## INTRODUCTION

Urological laparoscopic surgery has been steadily developing and is now a part of the routine care of many urological conditions. However, laparoscopic surgery poses specific challenges that require acquisition of difficult skills and lead to a steeper learning curve when compared with open surgery (1). Training in laparoscopic surgery has evolved since its inception in the 1980s with the creation of multiple simulation centers where surgeons can acquire skills outside of the operating room (2, 3).

Since the introduction of robotic surgical systems at the last century, the growth of robotic surgical practice in urology has been exponential (4). This growth, in the use of robotic platforms, has occurred without a simultaneous shift in the manner by which training takes place (5). Concerns exist both over the quality of robotic surgical training and the effect of robotic practice has had on urological training in general (6).

The objectives of this study are to undertake a census to determinate the current state of laparoscopic and robotic urological practice and to know the mains barriers to adequate practice in Brazil.

## MATERIALS AND METHODS

In August 2017, the department of minimally invasive surgery of the Brazilian Society of Urology (SBU) designed non-validated surveys based on the vast experience of the members of the department, consisting of an anonymous questionnaire with 15 questions prepared through the Google® Forms website (7). This method of questions was sent via internet through the mailing list of the SBU to all active members and a link to the survey website was set in the covering letter. Responses were taken using the automated process of the website and were kept open for 3 months.

A minimum sample was calculated so that the data could be significantly analyzed. The calculation was used for sampling a finite population, adopting the error of 5% and confidence interval of 95%, reaching the result of a minimum sample of 359 participants (8).

The mains topics evaluated in the survey were if the urologist performed minimally invasive surgery, how many they operated in average, which exact surgery they performed by pure laparoscopy or robotic, motive for not performing minimally invasive surgery, how long they were in the field, demographic information and if they would participate in hands-on courses. In some questions, participants were able to choose more than one response (Table-1).

**Table 1:** Questions and possible answers sent for SBU members.

Questions	Possible answers
Do you perform videosurgery?	Yes or No.
If yes, how many videosurgeries do you perform in a month? (average)	0-5 or 5-10 or >10
If laparoscopy is performed, which of the following do you do?*	Total and radical nephrectomy, Partial nephrectomy, Radical prostatectomy, Prostatectomy for benign prostatic hyperplasia (BPH), Cystectomy, Pyeloplasty, Sacrocolpopexy, Adrenalectomy, Others
If you answered no or consider doing a small number of procedures, what would be the reason?*	Lack of trained support staff (assistants, nursing, etc.), Lack of patients, Lack of practical experience in the method, Lack of equipment/infrastructure in the hospital I attend, Lack of theoretical knowledge about the method
Do you perform robotic surgery?	Yes or No.
If yes, how many robotic surgeries do you perform in a month? (average)	0-5 or 5-10 or >10
If robotic surgery is performed, which of the following do you do?*	Total and radical nephrectomy, Partial nephrectomy, Radical prostatectomy, Prostatectomy for BPH, Cystectomy, Pyeloplasty, Sacrocolpopexy, Adrenalectomy, Others
If you answered no or consider doing a small number of robotic procedures, what would be the reason?*	Lack of trained support staff (assistants, nursing, etc.), Lack of patients, Lack of practical experience in the method, Lack of equipment/infrastructure in the hospital I attend, Lack of theoretical knowledge about the method
How long have you been a professional urologist? (Years)	0-10 or 10-20 or >20
What region of Brazil do you perform your urological activity?	Northeast or Southeast or Mid-West or South or North.
Your area of activity is located in the interior or capital of the state?	Interior or Capital
What is the approximate number of inhabitants of the municipality in which you operate? (Thousands)	<100 or 100-500 or >500
Would you participate in theoretical and practical courses (hands-on and live surgeries) taught by national and international experts in your region?	Yes or No.
How far would you travel to participate in this type of course? (Km)	0-50 or 50-200 or >200
Give a suggestion of a city of your region to carry out a course of this type	Open question

*Participants were able to choose more than one response.

With these data, activities related to laparoscopy and robotic surgery of our urologists and the mains difficulties and barriers to minimally invasive practice were evaluated and we correlated the experience as urologist, the region in Brazil they worked and the area they were located with performing video surgery and robotic surgery using the Chi-square Test of independence.

All the answers were collected in the Google® Forms` database in the internet and the information was transferred to Microsoft® Excel. Subsequently, analysis was done using IBM SPSS Statistics for Windows, Version 22.0 (IBM, New York, USA). A p <0.05 was considered to indicate statistical significance.

## RESULTS

In all, 413 questionnaires were completed, sent and analyzed. The majority of the respondents were currently working in the Southeast region of Brazil (52.1%), followed of South region (20.3%), Northeast region (17.9%), Mid-west region (6.5%) and North region (3.1%). Urologists currently working in the capital corresponded to 55.7%, while 44.3% of the respondents were in the interior.

Considering an estimated total number of urologists with a specialist title in Brazil to be 5328 (9) and the 413 questionnaires analyzed, the overall urology specialist’s response rate was 7.75% and the 95% confidence interval (CI) was 7.03%-8.47%.

Among the respondents, 61.9% were working in cities with >500.000 inhabitants, 28.9% in cities with 100-500.000 inhabitants and 9.2% in cities with <100.000 inhabitants.

Most part of respondents had less than 10 years as urologists (50.9%) and experienced urologists were less common (23.2% between 10 to 20 years and 25.9% with more than 20 years).

In our survey, 75.5% of the surgeons performed laparoscopic surgery and only 12.8% robotic surgery. When asked about the number of laparoscopic surgeries performed per month, 61.8% were performing five procedures, 24.9% between five to ten surgeries and 13.2% more than 10 procedures. Among robotic surgeons, the number of monthly surgeries were 5 or less in 77.4%, between 5 to 10 in 14.5% and more than 10 in 8.1%.

**Figure 1:**
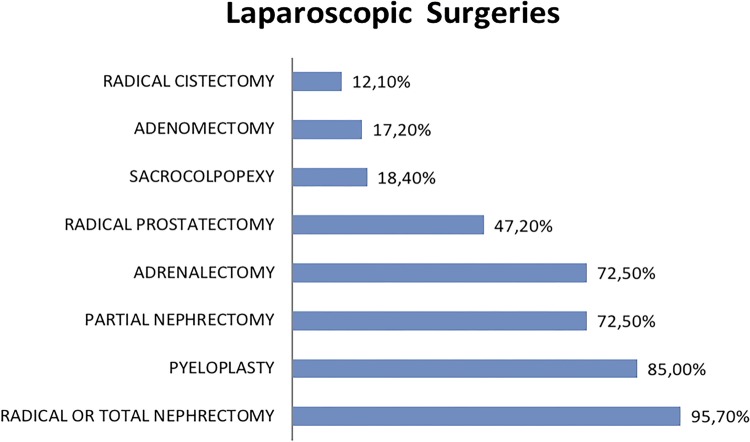
Surgeries performed by laparoscopic surgeons.

Including the laparoscopic procedures, the most frequently done is total or radical nephrectomy by 95.7% of respondents, followed by pyeloplasty (85.2%). Partial nephrectomy and adrenalectomy are performed by 72.5% of laparoscopic urologists. Less than half of laparoscopic urologists made radical prostatectomies (47.2%), sacrocolpopexy (18.4%), prostatectomy for BPH (17.2%) or radical cystectomy (12.1%) (Figure-1).

**Figure 2:**
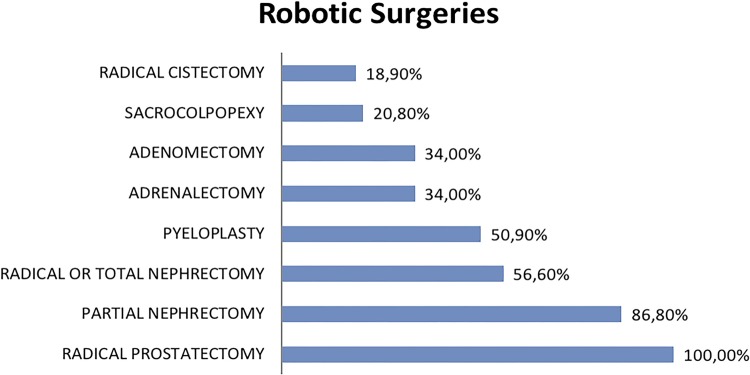
Surgeries performed by robotic surgeons.

All robotic surgeons inquired performed radical prostatectomy (100%) and 86.8%, partial nephrectomy. Also, urologists trained in robotic surgery responded that they performed total or radical nephrectomy (56.6%), pyeloplasty (50.9%), adrenalectomy (34%), prostatectomy for BPH (34%), sacrocolpopexy (20.8%) or, less commonly, radical cystectomy (18.9%) (Figure-2).

**Figure 3:**
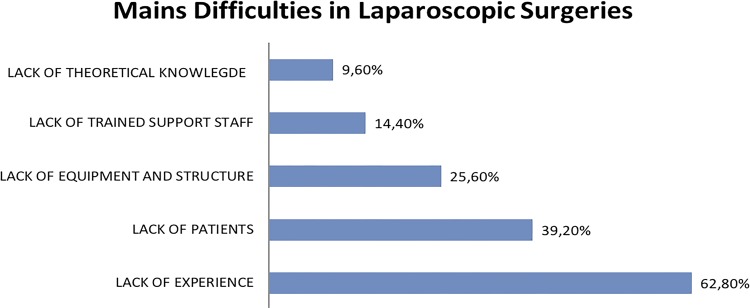
Mains difficulties in laparoscopic surgeries.

When inquired about the main difficulties for not executing or performing a small number of laparoscopic surgeries, the lack of practical experience on the method was pointed out in 62.8% of responders. Following, the lack of patients (39.2%), of equipment and structure (25.6%), of trained support staff (14.4%) and of theoretical knowledge (9.6%) were highlighted (Figure-3).

**Figure 4:**
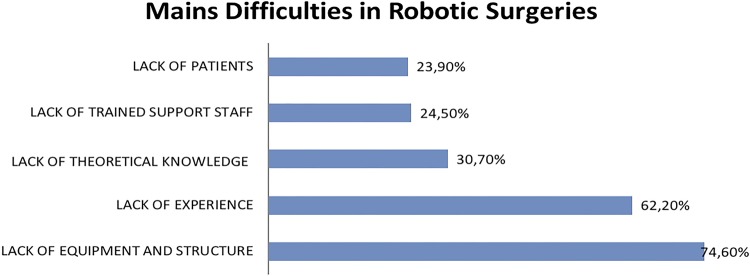
Mains difficulties in robotic surgeries.

Mains barriers and difficulties for not executing robotic surgery were the lack of equipment/infrastructure (74.6%), of experience in this modality (62.2%), of theoretical knowledge (30.7%), of trained support staff (24.5%) and lack of patients (23.9%) (Figure-4).

Among all respondents, 78% would participate in theoretical and practical courses (hands-on and live surgeries) taught by national and international experts.

A Chi-square test of independence was calculated comparing the time as they worked as urologists and if they performed laparoscopy which found a significant interaction (χ^2^(2)=20.576, p <0.05) and if they performed robotic surgery which was not significant (p=0.507).

A Chi-square test of independence was calculated comparing the region in our country they worked and if they performed laparoscopy which did not found a significant interaction (χ^2^(4)=5.870, p=0.209) and if they performed robotic surgery which was significant (χ^2^(4)=11.745, p=0.019).

A Chi-square test of independence was calculated comparing the area (capital or interior) they worked and if they performed laparoscopy which found a significant interaction (χ^2^(2)=34.749, p <0.05) and if they performed robotic surgery which was significant (χ^2^(2)=27.200, p <0.05).

## DISCUSSION

This study, for the first time, provides an overview of laparoscopic and robotic urological practice in Brazil. In our country, the first laparoscopic surgery was done in 1991 in Rio de Janeiro and the initial experience in robotic surgery took place at São Paulo in 2008 (9).

A significant number of responses were obtained and the distribution of the answers were very similar of that of the urologists of our country according to our Federal Medical Council (10). All 5328 Brazilian urologists are divided as follows: North region (4.3%), Northeast region (16.5%), Southeast region (52.2%), South (16.9%) and Mid-west region (10%) (10). While our responses were obtained from the following regions: North region (3.1%), Northeast region (17.9%), Southeast region (52.1%), South (20.3%) and Mid-west region (6.5%).

Our study has some limitations. Since it was an internet survey, a bias was introduced to those who regularly check and use e-mail. Urologists, who respond to e-mail surveys, may be more likely to embrace technological advances and answer questions involving new and minimally invasive techniques. Also, the survey was sent only to those who are members of the SBU.

Half of the urologists who participated of the census (50.2%) had less than 10 years of experience, 23.2% had a professional career between 10 to 20 years and 25.9% had more than 20 years. The median time of experience of all 5328 urologists in Brazil is 23.4 years according to our federal council (10).

Considering that laparoscopy was introduced more frequently in our resident’s practice in the 2000s, the years of experience as formed urologists might also have a significant consequence about questions of laparoscopic surgery, once urologists are more likely to perform laparoscopic procedures if they were trained during residency than if they had no experience (11, 12). The percentage of those who perform laparoscopic surgery is 75.5%, which demonstrates a strong implementation of this technique among the urologists of our country. Among these professionals, 56.4% have less than 10 years of experience, 22.7% have between 10 to 20 years and 20.8% have more than 20 years as specialists (p <0.05).

As robotic surgery was initiated relatively 10 years ago in Brazil, the training occurred outside public hospitals with residents, and more experienced urologists in private’s hospitals were able to train with the robotic platform (9). In our survey, between those who performed robotic surgery, 43.3% had less than 10 years of experience, 26.4% between 10 to 20 years and 30.1% more than 20 years. We demonstrate that the majority (56.5%) of robotic surgeons inquired in Brazil have more than 10 years as specialists (p=0.507).

Total or radical nephrectomy is the most performed procedure by urologic laparoscopists (95.7%) and partial nephrectomy which had an increasing number of indications, is less commonly performed (72.5%) probably due to major complexity in some cases. Total or radical robotic-assisted nephrectomy is performed only by 56.6% of the surgeons already trained and could be explained due to lesser complexity, high costs and similar oncological and functional results when compared to pure laparoscopic technique. Nevertheless, partial nephrectomy, which is theoretically more complex and has advantages with the robotic platform, is performed by 86.8% of robotic surgeons.

Perhaps, the more complex steps in pure laparoscopic radical prostatectomy might be the main cause of only 47.2% being able to perform this technique and, on the other hand, 100% of robotic surgeons perform the robotic radical prostatectomy (13).

The main limitation in performing laparoscopic procedures is, the lack of practical experience with the technique (62.8%). Other factors considered relevant were the lack of patients (39.2%) and of equipment and structure (25.6%). In Brazil, we have several difficulties in our public health system, like lack of equipment for minimally invasive surgery, infrastructure and trained teams, not only in the interior of the country but also in some less developed capitals.

On the other hand, when we observe robotics surgeries, the main barriers are availability and access to the robotic platform (74.6%), followed by the lack of experience (62.2%). The small number of robotic platforms, around 30, when the census was distributed between August and October of 2017 and the limitations imposed, by the hospitals, to practice, explain these related difficulties. Almost all robots in Brazil are situated at the capitals, except for one, in the interior of São Paulo, which reflects a bigger proportion of robotic surgeries performed in the capitals of the states of Brazil.

## CONCLUSIONS

At our country, we have specific difficulties on the development of minimally invasive technologies, mainly in some regions, including the lack of equipment, experience and trained support staff. There are still barriers like high costs of the robotic platform, few urologists able to practice on the robotic platform and inadequate medical compensation (some minimally invasive surgeries are not included in the medical insurance fee hall).
